# The Genetic Background Modulates the Evolution of Fluoroquinolone-Resistance in *Mycobacterium tuberculosis*

**DOI:** 10.1093/molbev/msz214

**Published:** 2019-09-18

**Authors:** Rhastin A D Castro, Amanda Ross, Lujeko Kamwela, Miriam Reinhard, Chloé Loiseau, Julia Feldmann, Sonia Borrell, Andrej Trauner, Sebastien Gagneux

**Affiliations:** 1 Swiss Tropical and Public Health Institute, Basel, Switzerland; 2 University of Basel, Basel, Switzerland

**Keywords:** *Mycobacterium tuberculosis*, antimicrobial resistance, evolution, fluoroquinolones, epistasis, mycobacteria, fitness

## Abstract

Fluoroquinolones (FQ) form the backbone in experimental treatment regimens against drug-susceptible tuberculosis. However, little is known on whether the genetic variation present in natural populations of *Mycobacterium tuberculosis* (*Mtb*) affects the evolution of FQ-resistance (FQ-R). To investigate this question, we used nine genetically distinct drug-susceptible clinical isolates of *Mtb* and measured their frequency of resistance to the FQ ofloxacin (OFX) in vitro. We found that the *Mtb* genetic background led to differences in the frequency of OFX-resistance (OFX-R) that spanned two orders of magnitude and substantially modulated the observed mutational profiles for OFX-R. Further, in vitro assays showed that the genetic background also influenced the minimum inhibitory concentration and the fitness effect conferred by a given OFX-R mutation. To test the clinical relevance of our in vitro work, we surveyed the mutational profile for FQ-R in publicly available genomic sequences from clinical *Mtb* isolates, and found substantial *Mtb* lineage-dependent variability. Comparison of the clinical and the in vitro mutational profiles for FQ-R showed that 51% and 39% of the variability in the clinical frequency of FQ-R *gyrA* mutation events in Lineage 2 and Lineage 4 strains, respectively, can be attributed to how *Mtb* evolves FQ-R in vitro. As the *Mtb* genetic background strongly influenced the evolution of FQ-R in vitro, we conclude that the genetic background of *Mtb* also impacts the evolution of FQ-R in the clinic.

## Introduction

Antimicrobial resistance (AMR) poses a major threat to our ability to treat infectious diseases (MacGowan 2008; Winston and Mitruka 2012). The rise of AMR is a complex phenomenon with a broad range of contributing socioeconomic and behavioral factors (Dalton et al. 2012; Merker et al. 2015; Alvarez-Uria et al. 2016; Eldholm et al. 2016; Shah et al. 2017). However, the emergence of AMR within any pathogen population is ultimately an evolutionary process (zur Wiesch et al. 2011; Hughes and Andersson 2017). This evolutionary process is influenced by multiple factors, including drug pressure and pathogen genetics. Firstly, the drug type and drug concentration can affect the nature and relative frequencies of AMR mutations observed in a given pathogen population (also known as the mutational profile for AMR) (Zhou et al. 2000; Ford et al. 2013; Lindsey et al. 2013; McGrath et al. 2014; Hughes and Andersson 2017; Huseby et al. 2017). Secondly, pathogen populations comprise genetically distinct strains, and this genetic variation may also influence AMR evolution (Fenner et al. 2012; Vogwill et al. 2014, 2016; Gagneux 2018). Different pathogen genetic backgrounds can have different baseline susceptibilities to a given drug (Ängeby et al. 2010; Coeck et al. 2016), which can consequently affect patient treatment outcomes (Colangeli et al. 2018). The genetic background has also been shown to modulate the acquisition and prevalence of AMR (Borrell and Gagneux 2009; Fenner et al. 2012; Ford et al. 2013; Wollenberg et al. 2017), the mutational profile for AMR (Fenner et al. 2012; Ford et al. 2013; Vogwill et al. 2014; Oppong et al. 2019), and the phenotypic effects of AMR mutations (Gagneux et al. 2006; Decuypere et al. 2012; Angst and Hall 2013; Vogwill et al. 2016). Studying the interplay between pathogen genetics and drug pressure is therefore important in understanding how to restrict the emergence of AMR in pathogen populations.

AMR in *Mycobacterium tuberculosis* (*Mtb*), the etiological agent of human tuberculosis (TB), is of particular importance. *Mtb* infections globally cause the highest rate of mortality due to a single infectious agent both in general, and due to AMR specifically (World Health Organization 2017). Although the genetic variation in *Mtb* is small compared with other bacterial pathogens (Comas et al. 2010; Gagneux 2018), several studies have shown that this limited genetic variation influences AMR phenotypes and prevalence (Gagneux et al. 2006; Zaczek et al. 2009; Fenner et al. 2012; Gagneux 2018). The global genetic diversity of *Mtb* comprises seven phylogenetic lineages (Comas et al. 2010; Gagneux 2018), and *Mtb* strains belonging to the Lineage 2 Beijing/W genetic background have repeatedly been associated with multidrug-resistant TB (MDR-TB; defined as an infection from an *Mtb* strain that is resistant to at least isoniazid and rifampicin) both in vitro and in clinical settings (Borrell and Gagneux 2009; Fenner et al. 2012; Ford et al. 2013; Merker et al. 2015; Wollenberg et al. 2017).

One strategy to reduce the emergence of AMR in *Mtb* is the development of new, shorter treatment regimens (Imperial et al. 2018; Vjecha et al. 2018). Many such experimental regimens use third- or fourth-generation fluoroquinolones (FQ) against drug-susceptible *Mtb* (Gillespie et al. 2014; Jindani et al. 2014; Merle et al. 2014; Imperial et al. 2018; Vjecha et al. 2018). However, FQs have long been integral to treating MDR-TB (Takiff and Guerrero 2011), and the previous use of FQs has led to the emergence of FQ-resistance (FQ-R) in clinical strains of *Mtb* (Takiff et al. 1994; Maruri et al. 2012; Shah et al. 2017). FQ-R is one of the defining properties of extensively drug-resistant TB (XDR-TB), and XDR-TB accounts for 8.5% of MDR-TB cases (World Health Organization 2017). Understanding how FQ-R is acquired in natural populations of *Mtb* may allow for the development of tools or strategies to mitigate further increases in FQ-R prevalence.

In *Mtb*, the sole target of FQ is DNA gyrase (Takiff et al. 1994; Zhou et al. 2000; Piton et al. 2010; Aldred et al. 2016; Blower et al. 2016). Consequently, clinically relevant FQ-R in *Mtb* is primarily due to a limited set of chromosomal mutations located within the “quinolone-resistance-determining region” (QRDR) of the *gyrA* and *gyrB* genes, which encode DNA gyrase (Takiff et al. 1994; Maruri et al. 2012; Wollenberg et al. 2017). No horizontal gene-transfer or plasmid-based resistance to FQ has been documented in *Mtb* (Boritsch et al. 2016; Gygli et al. 2017). Studying FQ-R evolution in *Mtb* populations thus provides a promising setting for elucidating how the genetic background may affect the emergence and maintenance of clinically relevant chromosomal AMR mutations in bacterial populations.

While a great deal of literature exists on the biochemical mechanisms leading to the FQ-R phenotype in *Mtb* (Zhou et al. 2000; Piton et al. 2010; Mustaev et al. 2014; Aldred et al. 2016; Blower et al. 2016), little is known on the evolutionary dynamics of FQ-R in different populations of *Mtb*. Given that antimicrobial regimens against *Mtb* infections use standardized, empirical dosing strategies (World Health Organization 2017), it is unclear whether different *Mtb* genetic backgrounds would acquire FQ-R at the same frequency when exposed to the same antimicrobial concentration. Whether the *Mtb* genetic background would also modulate the mutational profile for FQ-R, or the phenotypic effects of FQ-R mutations, is unknown. Such knowledge may provide insights on how to maintain or prolong the efficiency of FQs against different genetic variants of *Mtb* in the clinic.

In this study, we tested whether the *Mtb* genetic background plays a role in the evolution of FQ-R. We showed that the *Mtb* genetic background can lead to differences in the frequency of FQ-R emergence that span two orders of magnitude, as well as substantially modulate the mutational profile for FQ-R. We further demonstrated that the phenotypic effects of clinically relevant FQ-R mutations differed depending on the *Mtb* genetic background they were present in. Analysis of publicly available genomic sequences from clinical *Mtb* isolates also revealed a positive association between the FQ-R mutational profiles observed in vitro and the mutational profiles observed in the clinic. Taken together, we showed that the *Mtb* genetic background had a considerable role in evolution of FQ-R in the clinic.

## Results

### Frequency of Ofloxacin-Resistance in *Mtb* Is Strain-Dependent

**Fig. 1.**
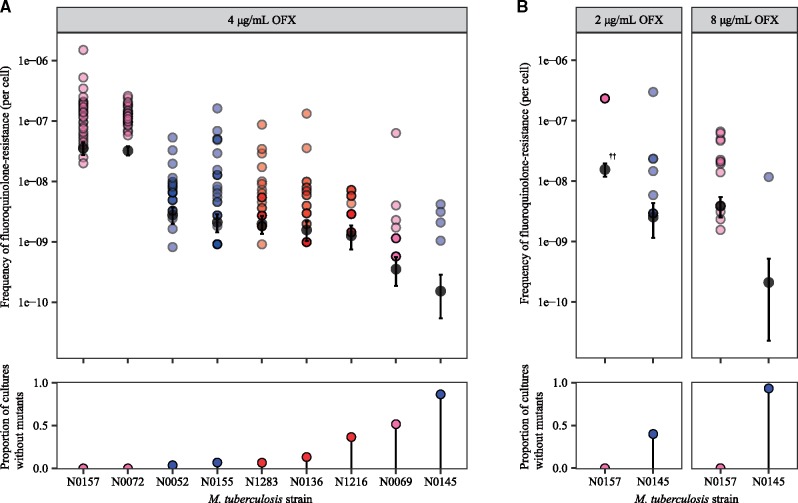
Variation in the frequency of ofloxacin-resistance between genetically distinct, wild-type *Mycobacterium tuberculosis* strains. (*A*) Frequency of ofloxacin-resistance at 4 µg/ml ofloxacin (OFX), as measured by fluctuation analysis. Top panel: colored points represent the frequency of ofloxacin-resistance per cell per parallel culture, with darker points representing multiple cultures with the same frequency. Colors denote the lineage that the *Mtb* strain belongs to (L1, pink; L2, blue; L4, red). Gray points represent the estimated number of drug-resistance mutations per cell per strain as calculated by MSS-MLE, while black bars denote the respective 95% confidence intervals. Bottom panel: the percentage of parallel cultures lacking OFX-resistant mutants. (*B*) Frequency of ofloxacin-resistance at 2 or 8 µg/ml OFX. ††Due to restrictions on the range of values that the MSS-MLE method is valid for (see Materials and Methods), the estimated number of drug-resistance mutations per cell for N0157 at 2 µg/ml OFX, as presented here, is an underestimate.

We first tested for whether the *Mtb* genetic background led to differences in the frequency of FQ-R acquisition. To do so, we performed a Luria–Delbrück fluctuation analysis on nine drug-susceptible and genetically distinct *Mtb* clinical strains belonging to Lineage 1 (L1), Lineage 2 (L2), and Lineage 4 (L4) (supplementary table S1, Supplementary Material online) (Luria and Delbrück 1943; Comas et al. 2010; Gagneux 2018; Borrell et al. 2019). We measured their frequency of resistance in vitro to the FQ ofloxacin (OFX), as OFX was used extensively to treat MDR-TB patients in the past (Takiff and Guerrero 2011). Given that anti-TB treatment regimens use standardized drug concentrations (World Health Organization 2017), we also measured the frequency of resistance to the same concentration of OFX (4 µg/ml) for all nine strains. We observed significant strain-dependent variation in the frequency of OFX-resistance (OFX-R) at 4 µg/ml, with the difference spanning two orders of magnitude (fig. 1*A*; *P *=* *2.2 × 10^−16^, Kruskal–Wallis). Several of the nine drug-susceptible *Mtb* strains contained missense substitutions in DNA gyrase that are not associated with FQ-R (supplementary table S2, Supplementary Material online) (Borrell et al. 2019). These mutations are phylogenetic markers that reflect the population structure of *Mtb* and cannot be avoided if strains from different *Mtb* lineages are used (Comas et al. 2010; Gagneux 2018). We found no evidence for any associations between the presence a given phylogenetic DNA gyrase missense mutation and the frequency of OFX-R acquired.

The concentration of the antimicrobial can affect the observed frequencies of AMR in *Mtb* (Zhou et al. 2000; Ford et al. 2013; McGrath et al. 2014). Therefore, we tested whether changing the selective concentration of OFX would affect the relative differences in strain-specific OFX-R frequencies. For the sake of simplicity, we tested only two strains, with each strain at the opposite extremes of the frequency of resistance to 4 µg/ml OFX, as shown in figure 1*A*: N0157 (high OFX-R frequency) and N0145 (low OFX-R frequency). We found that the frequency of OFX-R remained one to two orders of magnitude higher in N0157 than in N0145 across all the concentrations we tested (fig. 1*B*, *P *=* *2.46×10^−5^ for 2 µg/ml OFX, and *P *=* *4.03×10^−6^ for 8 µg/ml OFX, Wilcoxon rank-sum test). The N0157 strain had nearly confluent growth at 2 µg/ml OFX, which is the OFX concentration that has been shown to inhibit 95% of *Mtb* strains that have not been previously exposed to OFX, but does not inhibit *Mtb* strains that are considered resistant to OFX in the clinic (Ängeby et al. 2010; Coeck et al. 2016). This suggested that N0157 has low-level resistance to OFX, despite having no mutation in the QRDR. Meanwhile, at 8 µg/ml OFX, we observed only four resistant colonies for N0145 across all samples, with all colonies arising within the same culture.

The variation in OFX-R frequencies when selecting on the same concentration of OFX may be driven by several, nonexclusive biological factors. Firstly, the *Mtb* strains we tested may have different baseline DNA mutation rates. Secondly, the number and relative frequency of potential mutations that confer OFX-R may vary depending on the *Mtb* genetic background. Thirdly, the relative cost of OFX-R mutations may differ between *Mtb* genetic backgrounds. As the observed frequency of OFX-R in *Mtb* is likely the result from a combination of multiple factors, we took advantage of the fact that we had identified strains with a range of OFX-R frequencies. We selected three representative strains with significantly different OFX-R frequencies: N0157, N1283, and N0145. These strains had a high-, mid-, and low frequency of OFX-R, respectively (fig. 1*A*). We then explored the relative contributions of each biological factor listed above in driving the variation in OFX-R across genetically distinct *Mtb* strains.

### Mutation Rate Differences Do Not Drive the In Vitro Variation in OFX-R Frequency in *Mtb*

**Fig. 2.**
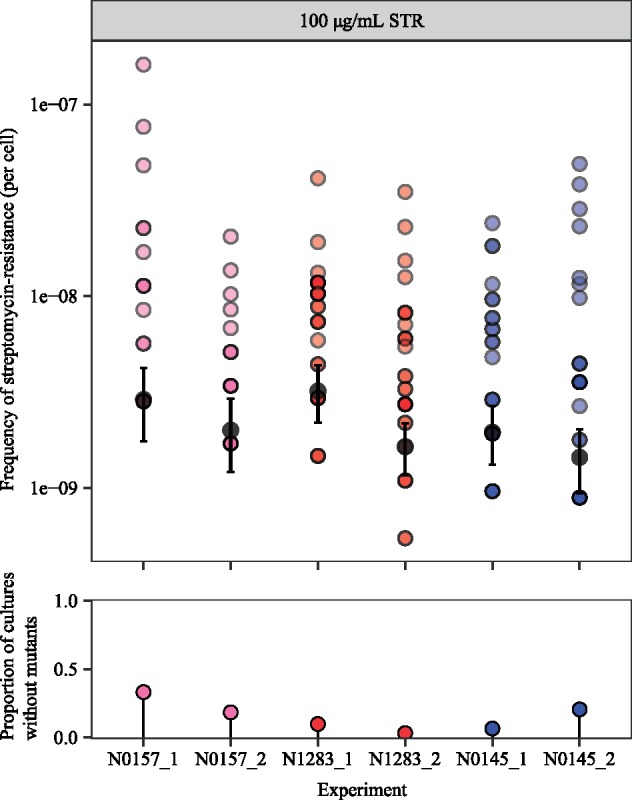
The frequency of streptomycin-resistance at 100 µg/ml streptomycin (STR) for wild-type N0157, N1283, and N0145 *Mycobacterium tuberculosis* strains, as measured by fluctuation analysis. Top panel: colored points represent the frequency of streptomycin-resistance per cell per parallel culture, with darker points representing multiple cultures with the same frequency. Colors denote the lineage that the *Mtb* strain belongs to (L1, pink; L2, blue; L4, red). Gray points represent the estimated number of drug-resistance mutations per cell per strain as calculated by MSS-MLE, while black bars denote the respective 95% confidence intervals. Bottom panel: the percentage of parallel cultures lacking STR-resistant mutants. Two biological replicates are presented for each *Mtb* strain, with each replicate identifier suffixed after the strain name.

We first tested for the presence of differential mutation rates between our panel of *Mtb* strains in figure 1*A*. Mutations in *dnaE*, which encodes the replicative DNA polymerase and serves as the major replicative exonuclease in *Mtb*, have been shown to confer a hypermutator phenotype in *Mtb* in the absence of environmental stress (Rock et al. 2015; Baños-Mateos et al. 2017). While *dnaE* mutations were present in the genomic data of our panel of drug-susceptible *Mtb* strains (supplementary table S2, Supplementary Material online) (Borrell et al. 2019), none was in the polymerase and histidinol phosphatase domain of DnaE, the region where mutations would impart a hypermutator phenotype (Rock et al. 2015; Baños-Mateos et al. 2017). We did not test for the presence of *dnaE* mutations in the resistant colonies following the fluctuation analysis, as we reasoned that the likelihood of gaining both a *dnaE* and a *gyrA* double mutation within this relatively short period is extremely low as to be considered negligible. To test for mutation rate variation in vitro, we again conducted a fluctuation analysis on N0157, N1283, and N0145 (the high-, mid-, and low-frequency OFX-R strains, respectively), but used streptomycin (STR; 100 µg/ml) instead of OFX. We hypothesized that if the frequency of OFX-R is driven by differential mutation rates, then we should expect similar differences in the frequency of STR-resistance (STR-R). However, we observed no evidence for differences in the frequency of STR-R between the strains tested (fig. 2, *P *=* *0.135, Kruskal–Wallis; supplementary table S3, Supplementary Material online). This suggested that the observed differences in frequency of resistance are specific to OFX, and that there are limited, if any, inherent differences in mutation rates between the *Mtb* strains tested.

### Mutational Profile for OFX-R Is Highly Strain-Dependent

**Fig. 3.**
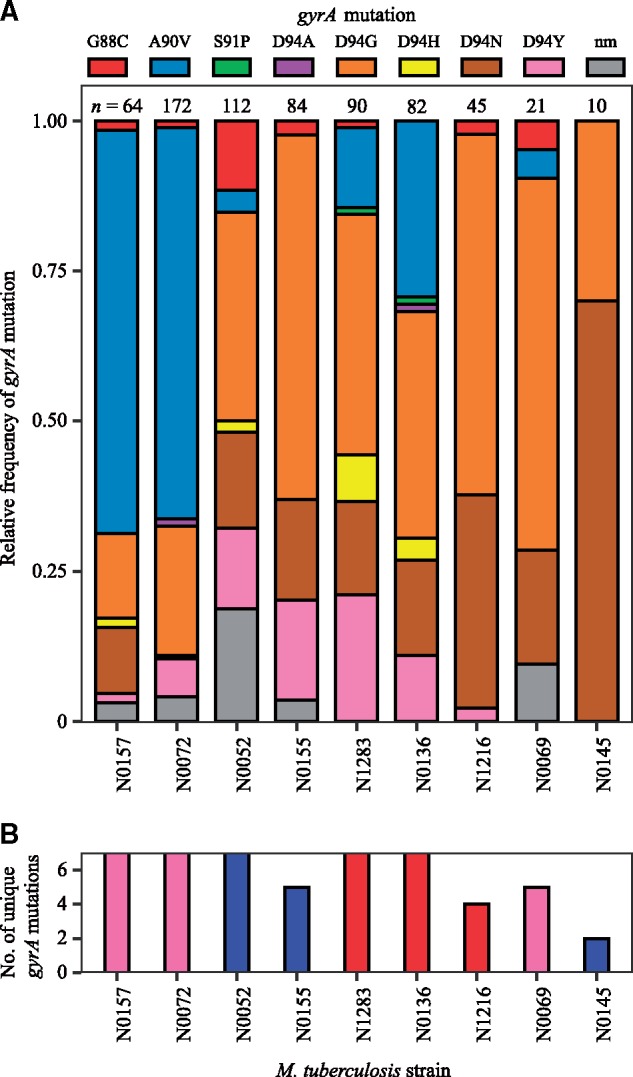
Variation in the mutational profile for ofloxacin-resistance after fluctuation analyses using nine genetically distinct *Mycobacterium tuberculosis* strains. (*A*) Mutations in the quinolone-resistance-determining region (QRDR) of *gyrA* were analyzed in 680 ofloxacin (OFX)-resistant colonies from the fluctuation analysis performed in figure 2*A* (nm, no identified QRDR *gyrA* mutations). Strains are ordered left to right based on their frequency of OFX-resistance at 4 µg/ml OFX. Numbers of colonies analyzed per strain are reported directly above each column. (*B*) The number of unique QRDR *gyrA* mutations per *Mtb* strain for OFX-resistance. Bar colors denote the *Mtb* lineage the strain belongs to (L1, pink; L2, blue; L4, red).

We next determined the mutational profile for OFX-R for each strain used in the fluctuation analysis at 4 µg/ml OFX (fig. 1*A*). The QRDR mutations in 680 *gyrA* and 590 *gyrB* sequences were identified in the resistant colonies. We observed that *gyrA* mutations made up 99.7% of the QRDR mutations observed (645 *gyrA* mutations, 2 *gyrB* mutations), and no QRDR double mutants were present (supplementary fig. S1 and tables S4 and S5, Supplementary Material online). The mutational profiles for OFX-R were also highly strain-specific (fig. 3*A*, *P *=* *5.00 × 10^−4^, Fisher’s exact test). Specifically, the GyrA A90V mutation was most prevalent in the high-frequency OFX-R strains, while GyrA D94G was most prevalent in all other strains. There was also a slight trend showing that strains with a greater number of unique *gyrA* mutations present also had higher rates of OFX-R (figs. 1*A* and 3*B*).

**Fig. 4.**
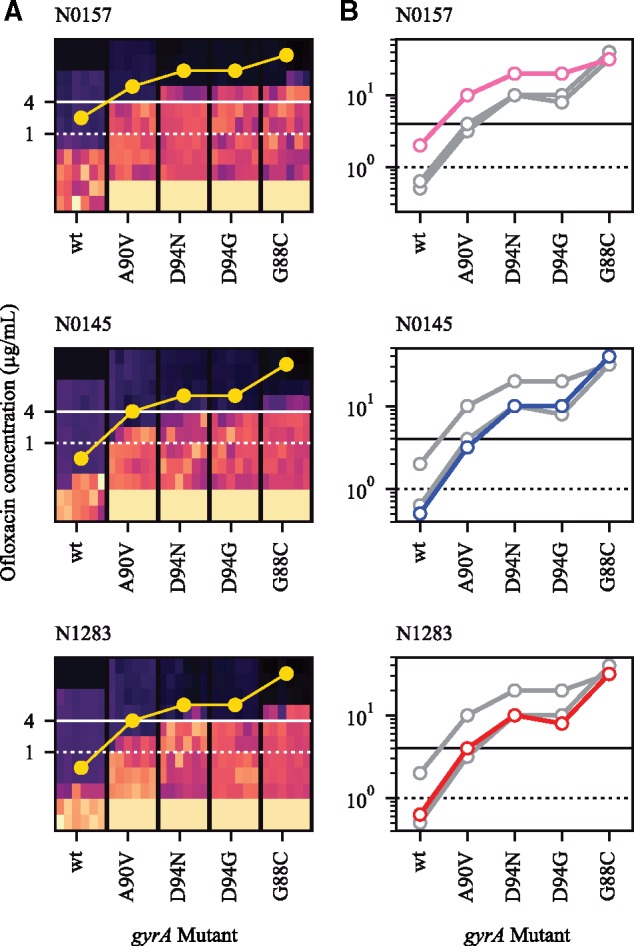
The *Mycobacterium tuberculosis* genetic background modulates the ofloxacin (OFX) minimum inhibitory concentration (MIC). (*A*) Heat-map of OFX-susceptibility via Alamar Blue assay for *gyrA* mutant strains of *Mtb*, as well as their wild-type ancestor, in three genetic backgrounds (N0157, N0145, or N1283). Light areas represent growing cultures, while dark areas represent nongrowing cultures. Yellow points represent estimates for OFX MIC (≥95% reduction in fluorescence). Areas of solid black colors (at 16+ μg/ml OFX for wild-type) and solid light beige colors (at <0.125 μg/ml OFX for mutants) were not measured and colored in for illustrative purposes. (*B*) OFX MIC estimates for each strain per genetic background, superimposed. Colored points and lines represent MIC measurements for highlighted genetic background, with the line color denoting the lineage that the strain belongs to (L1, pink, L2, blue, L4, red). Gray points and lines represent the other two genetic backgrounds. Solid horizontal black line denotes 4 μg/ml OFX, while dashed horizontal black line denotes 1 μg/ml OFX.

The strain-dependent variation in the mutational profile for OFX-R may be due to *gyrA* mutations conferring different resistance levels depending on the *Mtb* strain they are present in. To test this hypothesis, we first isolated OFX-R mutants carrying one of four possible GyrA mutations (G88C, A90V, D94G, or D94N) in the three strains used in figure 2: N0157, N1283, and N0145. The OFX MIC was determined for each of the 12 OFX-R mutant strains, along with their respective wild-type ancestors. We found that each parental wild-type strain had different susceptibilities to OFX, with N0157, N1283, and N0145 having OFX MICs of 2, 0.6, and 0.5 µg/ml, respectively (fig. 4*A* and supplementary table S6, Supplementary Material online). This was consistent with the fluctuation analysis results shown in figure 1*B*. Furthermore, we observed that the OFX MIC conferred by a given *gyrA* mutation varied depending on the strain it was present in (fig. 4*B* and supplementary table S6, Supplementary Material online). For example, mutants in the N0157 strain generally had higher OFX MICs than mutants in either the N0145 or N1283 strains. The only mutation that deviated from this trend was GyrA G88C, which conferred a higher OFX MIC when in the N0145 strain. Notably, the GyrA A90V mutation conferred a resistance level ≥4 µg/ml OFX in the N0157 and N1283 strains, but not in N0145. This was consistent with the presence of GyrA A90V in the OFX-R mutational profile for N0157 and N1283, but not in N0145, in the fluctuation analysis using 4 µg/ml OFX (figs. 1*A* and 3). In summary, the differences in OFX MIC reflected the strain-dependent mutational profiles for OFX-R in *Mtb*, as expected.

### Fitness of OFX-R Mutations Are Associated with Their Relative Frequency In Vitro

**Fig. 5.**
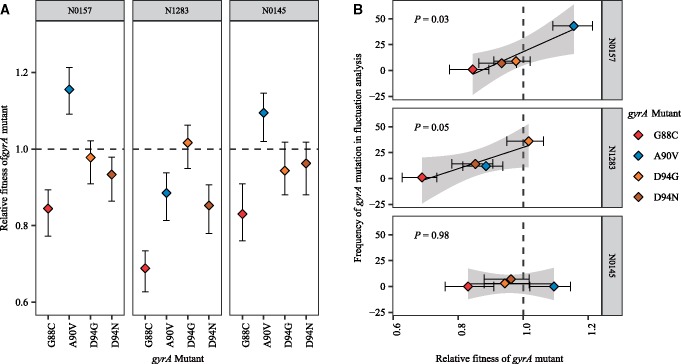
The *Mycobacterium tuberculosis* genetic background modulates the fitness effect of fluoroquinolone-resistance mutations. (*A*) Fitness of ofloxacin-resistant *Mtb* strain with specified *gyrA* mutation relative to the fitness of their respective wild-type ancestral strain. Fitness was measured by cell growth assay in antibiotic-free conditions. Ancestral strain per *gyrA* mutant is indicated in the gray bar above each panel. (*B*) Association between the relative fitness of specified *gyrA* mutant and their absolute frequency after the fluctuation analysis performed in figure 1*A* and as reported in figure 3*A*, in three genetic backgrounds (N0157, N1283, and N0145).

While the OFX MICs may determine which mutations may be observed in a fluctuation analysis, it is not the sole parameter to influence the OFX-R mutational profile for a given strain. We found that while the same *gyrA* mutation can be observed in two different *Mtb* strains, their relative frequencies may vary (fig. 3). This variation may be due to the fitness of a given *gyrA* mutant being different across genetic backgrounds. To test this hypothesis, we used cell growth assays in antibiotic-free conditions to measure the in vitro fitness of our panel of OFX-R mutants relative to their respective parental wild-type ancestors. We observed that the relative fitness of the OFX-R mutants was modulated by both the *gyrA* mutation and the *Mtb* strain they were present in (fig. 5*A*; supplementary figs. S2 and S3 and table S7, Supplementary Material online). Furthermore, there was a positive association between the fitness of a given *gyrA* mutation with its relative frequency in the fluctuation analysis for the N0157 and N1283 strains (fig. 5*B*, *P *=* *0.03 for N0157, *P *=* *0.05 for N1283). There was no evidence of an association in the N0145 background due to the lack of GyrA G88C and A90V mutants in its fluctuation analysis.

The results from figures 4 and 5, as well as the apparent lack of mutation rate differences between our strains (fig. 2), suggested that differential mutational profiles were an important contributor in the variation in OFX-R frequency in *Mtb*. These mutational profile differences appear to be driven by the *Mtb* genetic background’s effect on both the MIC and the relative fitness cost of OFX-R mutations. We next explored whether these in vitro results would be relevant in clinical settings.

### Mutational Profile for FQ-R In Vitro Reflects Clinical Observations

**Fig. 6.**
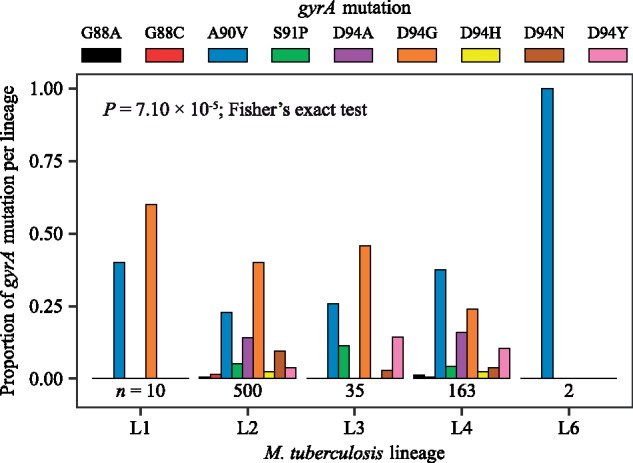
Mutational profile for fluoroquinolone-resistance *gyrA* mutations is lineage-specific in clinical isolates of *Mycobacterium tuberculosis*. An initial data set consisting of 3,450 genomes with confirmed MDR-TB mutations were surveyed. About 854 genomes were identified as fluoroquinolone-resistant, with 848 of these genomes containing at least one *gyrA* mutation. Only fixed fluoroquinolone-resistance mutations in the *gyrA* gene are enumerated here (*n* = 710). No fixed mutations were observed in Lineage 5 strains. Numbers of genomes analyzed per lineage are presented directly below their respective bar graph.

To explore the clinical relevance of our in vitro work, we surveyed the FQ-R mutational profile from publicly available *Mtb* genomes obtained from clinical isolates. FQs are generally used for treatment against MDR-TB (World Health Organization 2017). While it is unclear whether resistance mutations for isoniazid (INH) and/or rifampicin (RIF) predispose a strain to become FQ-R, the prevalence of FQ-R is heavily biased toward MDR-TB strains due to treatment practices. We therefore based our analyses on a collated data set of 3,450 publicly available MDR-TB genomes (supplementary table S8, Supplementary Material online), which we confirmed to be MDR-TB based on the presence of known INH-resistance (INH-R) and RIF-resistance (RIF-R) mutations. This data set provided a reasonable sampling of the overall genetic diversity of *Mtb*, as six of the seven known phylogenetic *Mtb* lineages were represented (Lineages 1–6) (Comas et al. 2010; Gagneux 2018). We catalogued their FQ-R mutational profiles, and found 950 FQ-R mutations in 854 genomes (supplementary tables S9 and S10, Supplementary Material online), showing that multiple FQ-R mutations may be present in the genome of a single *Mtb* clinical isolate. The frequency of FQ-R differed between lineages, with the highest frequencies present in L2 and L4 strains (supplementary table S9, Supplementary Material online, *P *<* *2.20 × 10^−16^, Chi-square Goodness of Fit Test). Moreover, we noticed a lineage-dependent mutational profile for FQ-R (fig. 6, *P *=* *7.10 × 10^−5^, Fisher’s exact test; supplementary tables S10 and S11, Supplementary Material online). For example, while the GyrA D94G mutation was most prevalent in strains belonging to L1, L2, and Lineage 3 (L3), the GyrA A90V mutation was most prevalent in L4 and Lineage 6 (L6).

**Fig. 7.**
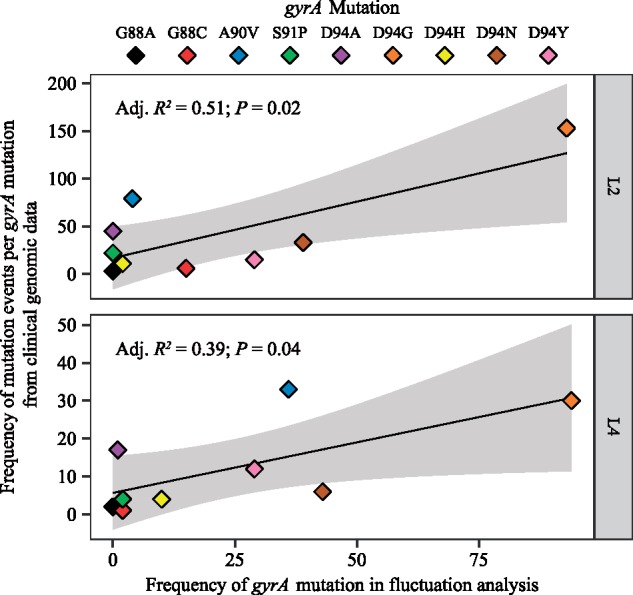
Association between the clinical frequency of mutation events of each fluoroquinolone-resistance (FQ-R) *gyrA* mutations with their respective in vitro frequencies among *Mycobacterium tuberculosis* strains belonging to either the L2 or L4 lineages. Mutation events per FQ-R *gyrA* mutation were enumerated from an initial data set of 3,450 genomes with confirmed MDR-TB mutations. Each unique and fixed FQ-R *gyrA* mutation present per transmission cluster (cutoff=12 SNPs average distance), as well as each fixed FQ-R *gyrA* mutation present in nonclustered genomes, were counted as independent mutation events. The in vitro frequencies of FQ-R *gyrA* mutations presented here are the same as in figure 3*A*, grouped by lineage.

We observed that the mutational profile for FQ-R in the fluctuation analysis experiments mimicked published clinical data. Firstly, *gyrA* mutations made up the large majority of FQ-R mutations in vitro (fig. 3 and supplementary tables S4 and S5, Supplementary Material online) and 944 out of the 950 QRDR mutations in the clinic (99.6%; supplementary table S10, Supplementary Material online). We then tested whether the *Mtb* genetic background had an impact on FQ-R *gyrA* mutational profiles in the clinic as it did in vitro. However, transmission events can modulate the frequency of FQ-R *gyrA* mutations in the clinic, but not in a fluctuation analysis. If each genome from the clinical data is treated as an independent event, then number of FQ-R *gyrA* mutation events in the clinic would be overestimated compared with the number of mutation events in a fluctuation analysis. Therefore, rather than directly compare the frequency of *gyrA* mutations from the OFX-R mutational profile in figure 3 to the absolute frequency of *gyrA* mutations in the genomic data survey, we instead compared the in vitro frequency of *gyrA* mutations in figure 3 to the frequency of mutation events per *gyrA* mutation in the genomic data. To do so, we first used differences in the number of single-nucleotide polymorphisms (SNPs) as a measure of genetic distance between two genomes, then defined transmission clusters within the 3,450 MDR-TB genomes via a conservative cutoff of 12 SNPs average distance (Walker et al. 2013). Each unique and fixed FQ-R *gyrA* mutation present per transmission cluster, as well as each fixed FQ-R *gyrA* mutations present in nonclustered genomes, were counted as independent mutation events. We limited our analysis to L2 and L4 strains, as these two lineages had the highest clinical frequencies of FQ-R. We observed that the profile for FQ-R *gyrA* mutation events in L2 strains differed significantly to L4 strains (supplementary fig. S5, Supplementary Material online, *P *=* *0.02, Fisher’s exact test; supplementary table S12, Supplementary Material online). Furthermore, there was a positive association between the frequency of a given FQ-R *gyrA* mutation in our fluctuation analysis compared with the frequency of its mutation event in the clinic for both L2 and L4 strains (fig. 7, *P = *0.02 for L2, *P *=* *0.04 for L4). Based on the adjusted *R*^2^ values, 51% of the variability in the clinical frequencies of FQ-R *gyrA* mutation events in L2 strains and 39% of the variability in L4 strains can be attributed to how FQ-R evolves in *Mtb* in vitro. As the in vitro evolution of FQ-R is itself modulated by the *Mtb* genetic background, this provided evidence for the *Mtb* genetic background’s role in the evolution of FQ-R in the clinic.

## Discussion

Overall, we illustrate the *Mtb* genetic background’s considerable role in the evolution of resistance to FQs, a clinically important antimicrobial. We first explored whether the genetic variation among natural populations of *Mtb* can influence FQ-R evolution in vitro. Specifically, considering that *Mtb* treatment regimens are based on standardized antimicrobial concentrations (World Health Organization 2017), we tested whether different genetic variants of *Mtb* would acquire FQ-R at the same frequency when exposed to the same concentration of FQ. Fluctuation analysis on nine, genetically distinct, drug-susceptible *Mtb* strains showed that the genetic background can have a drastic effect on the rate of OFX-R acquisition when using the same concentration of OFX (fig. 1). Our results provide the first evidence showing that the *Mtb* genetic background can modulate the frequency of FQ-R acquisition.

However, the effect of the *Mtb* genetic background on AMR frequencies observed here in the context of OFX-R differed from those reported in previous work focusing on other antibiotics. Past literature has focused on the positive association between MDR-TB and L2 Beijing (Borrell and Gagneux 2009; Fenner et al. 2012; Merker et al. 2015; Eldholm et al. 2016). Initial genetic analysis on a global collection of strains showed that mutations in DNA repair genes were associated with being MDR-TB, and that these mutations were specific to L2 Beijing isolates (Rad et al. 2003). The authors thus hypothesized that L2 Beijing strains may have a hypermutator phenotype, which would lead to higher rates of AMR mutations acquisition (Rad et al. 2003). Based on this L2 Beijing hypermutator hypothesis, one would expect that L2 Beijing strains would also show higher frequencies of FQ-R. However, this was not the case in our fluctuation analysis for OFX-R, as one of our L2 Beijing strains (N0145) repeatedly acquired the lowest frequency of OFX-R (fig. 1). Moreover, we saw minimal, if any, DNA base-pair mutation rate differences between three *Mtb* strains (one of which was L2 Beijing) with different in vitro OFX-R frequencies (fig. 2). Our results therefore contradict the L2 Beijing hypermutator hypothesis. Published experimental work have also provided varying results. Initial fluctuation analyses showed no difference in the frequency of RIF-R in L2 Beijing strains compared with non-L2 Beijing strains (Werngren and Hoffner 2003). In contrast, a fluctuation analysis performed by Ford et al. (2013) showed that L2 Beijing strains had higher frequencies of resistance for INH, RIF, and ethambutol compared with L4 strains, even after correcting for differences in AMR mutational profiles. Lastly, a more recent fluctuation analysis using the same concentrations of INH or RIF as Ford et al. showed that while different *Mtb* strains had different frequencies of INH-R, a L2 Beijing strain did not have higher frequencies of INH-R nor RIF-R compared with non-L2 Beijing strains (Carey et al. 2018). Although diverging in their results, these in vitro and genetic studies, together with the study conducted here, highlight the importance of the genetic background when testing for the frequency of AMR in *Mtb*. Furthermore, these results show that differential DNA mutation rates is not the only parameter relevant in determining the frequency of FQ-R in *Mtb*.

If DNA mutation rates do not contribute to the variation in OFX-R frequency, we hypothesized that differences in the phenotypic effects of OFX-R mutations, and their consequent effect on the mutational profiles for OFX-R, may be important contributors. By sequencing the QRDR from resistant colonies in our OFX fluctuation analysis, we observed strain-specific patterns in the mutational profiles for OFX-R (fig. 3). This suggested that the mutational profile for FQ-R is not only a function of the FQ type and concentration (Zhou et al. 2000; Malik et al. 2010, 2016; Huseby et al. 2017) but that epistatic interactions between a given FQ-R mutation and the genetic background may also play a role. Similar epistasis between the phenotype of a given AMR mutation and the genetic background have been observed in other bacteria. For example, a given RIF-R *rpoB* mutation can confer differential MIC and fitness costs depending on the genetic background it occurred in, or on the presence of other AMR mutations, in *Escherichia* *coli* (Angst and Hall 2013), *Pseudomonas* spp. (Vogwill et al. 2014, 2016), *Mycobacterium* *smegmatis* (Borrell et al. 2013), and *Mtb* (Gagneux et al. 2006; Zaczek et al. 2009). In line with these previous studies, we found that the OFX MIC and the fitness effect conferred by a given *gyrA* mutation varied significantly depending on the *Mtb* genetic background they occur in (figs. 4 and 5*A*; supplementary table S6, Supplementary Material online). These results suggest that epistasis plays a role in determining the strain-dependent OFX-R frequencies and mutational profiles observed during our fluctuation analyses (figs. 3 and 5*B*).

The epistasis between the *Mtb* genetic background and FQ-R mutations may have clinical consequences. A recent study has shown that drug-susceptible *Mtb* strains with higher MICs to INH and RIF were associated with increased risk of relapse following first-line treatment (Colangeli et al. 2018). FQ-R *gyrA* mutations that confer higher MICs, such as any *gyrA* mutation in codon D94 except for D94A, have also been associated with poorer treatment outcomes in MDR-TB patients (Rigouts et al. 2016; Farhat et al. 2017). Considering our observation that the *Mtb* genetic background affected both the OFX MICs and OFX-R mutational profiles (figs. 3 and 4; supplementary tables S4–S6, Supplementary Material online), the genetic background may therefore contribute to differences in patient treatment outcomes when using FQs as first-line drugs.

Using publicly available genomic data from *Mtb* clinical isolates, we observed significant lineage-dependent variation in the frequency of and mutational profiles for FQ-R (fig. 6). As expected, the vast majority of FQ-R mutations were observed in *gyrA* (supplementary tables S10 and S11, Supplementary Material online) (Takiff et al. 1994; Zhou et al. 2000; Piton et al. 2010; Maruri et al. 2012; Aldred et al. 2016; Blower et al. 2016; Wollenberg et al. 2017). FQ-R was also most frequent in L2 and L4. This was also as expected, as strains from the L2 Beijing sublineage are known to associate with MDR-TB (Borrell and Gagneux 2009; Fenner et al. 2012; Merker et al. 2015; Wollenberg et al. 2017), while L4 strains are the most prevalent globally, including in regions classified as high burden for TB (Stucki et al. 2016; World Health Organization 2017; Brynildsrud et al. 2018; Gagneux 2018). Consequently, strains from L2 and L4 would be more exposed to FQs, leading to the higher FQ-R frequencies observed in these two lineages. Furthermore, we observed that more than half of the variability in the clinical frequency of FQ-R *gyrA* mutation events in L2 strains can be explained by how *Mtb* evolves in vitro (fig. 7). However, the in vitro FQ-R evolution could only account for 39% of the variability for the frequency of FQ-R *gyrA* mutation events in clinical L4 strains. This suggested that while the *Mtb* genetic background can influence the evolution of FQ-R in the clinic, other factors (which may be independent of the *Mtb* genetic background) likely played strong roles as well. Epidemiological factors including socioeconomic disruptions, health system inefficiencies, and human behavior are well known risk factors for the emergence and transmission of AMR in *Mtb* (Dalton et al. 2012; Merker et al. 2015; Alvarez-Uria et al. 2016; Eldholm et al. 2016; Shah et al. 2017). Meanwhile, biological factors not explored in this study, such as antibiotic type and concentration (Zhou et al. 2000; Ford et al. 2013; Lindsey et al. 2013; McGrath et al. 2014; Mustaev et al. 2014; Malik et al. 2016), pharmacodynamic and pharmacokinetic features (Pienaar et al. 2017; Sarathy et al. 2018), and the selective pressure of the host immune system (Handel et al. 2009), may also influence the evolution of FQ-R.

How the genetic background modulates FQ-R evolution in the clinic may also differ between different bacterial pathogens. In contrast to *Mtb* where DNA gyrase is the sole target for FQs, Gram-negative bacteria such as *E. coli* and *Salmonella* have two targets for FQs: DNA gyrase and topoisomerase IV (Hooper 1999). In *E. coli*, the evolutionary trajectory toward high-level FQ-R generally involves the stepwise acquisition of FQ-R mutations in either DNA gyrase or topoisomerase IV (Huseby et al. 2017). Therefore, the genetic background of Gram-negative bacteria may modulate the phenotypes of FQ-R mutations not only in DNA gyrase but in topoisomerase IV as well. Nevertheless, a recent study has shown that a common FQ-R mutation in Gram-negative bacteria, GyrA S83L, confers different phenotypes depending on whether it is present in *E. coli* or in *Salmonella* (Apjok et al. 2019). This suggests that the genetic background of Gram-negative bacteria may affect the evolution of FQ-R in the clinic. This type of epistasis is also not restricted to bacteria. The genetic background modulated the phenotypes of AMR mutations in the protozoan parasite *Leishmania donovani* (Decuypere et al. 2012), and the phenotypes of mutations in yeast when exposed to different environments, including antimicrobial exposure (Mullis et al. 2018). Thus, while the mode of epistasis between the genetic background and the phenotypes of mutations may differ in different organisms and environments, published work and the results of our study provide compelling evidence that this epistasis is a major factor in the evolution of AMR in both prokaryotic and eukaryotic organisms.

Our study is limited by the fact that our survey of clinical FQ-R frequencies involved a genomic data set that was sampled by convenience. This data set was used due to its public availability, and may not be fully representative of FQ-R frequencies in *Mtb* populations. We noted that lineage-specific frequencies of FQ-R were likely biased due to the overrepresentation L2 and L4 strains. Thus, to acquire a better understanding on which FQ-R mutations appeared and at what frequency they occurred at in different *Mtb* lineages, either more genomes from clinical isolates from other *Mtb* lineages must be made available, or a population-based study must be undertaken, preferably in a high-burden MDR-TB region.

Exposure to quinolones have been shown to lead to SOS response-mediated mutagenesis, which can increase the rate of AMR acquisition, including resistance to quinolones themselves (Cirz et al. 2005; Malik et al. 2010; Frenoy and Bonhoeffer 2018). Therefore, the strain-dependent OFX-R acquisition rates (fig. 1) may be due to strain-dependent differences in the magnitude of quinolone-induced mutagenesis. We did not explicitly test for this possibility. However, phylogenetic SNPs present in SOS response-related genes may lead to strain-dependent differences in quinolone-induced mutagenesis, and we observed no such SNPs present across our panel of drug-susceptible *Mtb* strains (supplementary table S2, Supplementary Material online) (Borrell et al. 2019). Thus, we observed no genetic evidence for strain-specific SOS response-mediated mutagenesis. Furthermore, in *E. coli*, quinolone-induced quinolone-resistant mutations may only be observed after 5 days of incubation with quinolones, which is equivalent to >220 generations for wild-type *E. coli* (Cirz et al. 2005; Fujikawa and Morozumi 2005). Meanwhile, our wild-type *Mtb* strains were incubated for 40 generations at most in the presence of OFX (see Materials and Methods and supplementary table S7, Supplementary Material online), making the likelihood of observing OFX-induced OFX-R mutants in our in vitro system extremely low.

Another limitation of our study is that fluctuation analyses only model AMR emergence. Long-term population dynamics also play an important role in AMR evolution (zur Wiesch et al. 2011; Lindsey et al. 2013; Huseby et al. 2017). For example, population bottleneck events modulate AMR evolution during serial transfer experiments (Comas et al. 2012; Barrick and Lenski 2013; Vogwill et al. 2016; Huseby et al. 2017), and have also been hypothesized to strongly influence *Mtb* evolution in the clinic (Hershberg et al. 2008). Thus, modeling FQ-R evolution in *Mtb* in epidemiological settings would benefit from the use of some measure of long-term population dynamics and between-host transmission. Nevertheless, the fitness of AMR mutants is an important factor in determining its evolutionary fate (zur Wiesch et al. 2011; Angst and Hall 2013; Barrick and Lenski 2013; Lindsey et al. 2013; Hughes and Andersson 2017; Huseby et al. 2017) and its potential for between-host transmission (Comas et al. 2012; de Vos et al. 2013). Considering that the *Mtb* genetic background modulated the fitness effect of FQ-R mutations (fig. 5 and supplementary table S7, Supplementary Material online), the genetic background may modulate how likely FQ-R mutants transmit between patients.

In conclusion, we illustrate how the genetic variation present in natural populations of *Mtb* modulates FQ-R evolution. Considering the nonrandom geographic distribution of different *Mtb* genetic variants (Comas et al. 2010; Gagneux 2018), our work suggests that there may be regional differences in the rate of FQ-R emergence and FQ-R prevalence when using FQs as a first-line drug. We therefore highlight the importance of standing genetic variation in determining how FQ-R evolves in *Mtb* and, in general, how AMR evolves in pathogens.

## Materials and Methods

### Collection of Drug-Susceptible Clinical Isolates of *Mtb* Strains for In Vitro Studies

We used nine genetically distinct *Mtb* strains, with three strains from each of the following *Mtb* lineages: Lineage 1 (L1; also known as the East-Africa and India Lineage), Lineage 2 (L2; the East Asian Lineage), and Lineage 4 (L4; the Euro-American Lineage) (Comas et al. 2010; Gagneux 2018). All strains were previously isolated from patients, fully drug-susceptible, and previously characterized by Borrell et al. (2019) (supplementary table S1, Supplementary Material online).

Prior to all experimentation, starter cultures for each *Mtb* strain were prepared by recovering a 20 μl aliquot from frozen stocks into a 10 ml volume of Middlebrook 7H9 broth (BD), supplemented with an albumin (Fraction V, Roche), dextrose (Sigma–Aldrich), catalase (Sigma–Aldrich), and 0.05% Tween 80 (AppliChem) (hereafter designated as 7H9 ADC). These starter cultures were incubated until their optical density at wavelength of 600 nm (OD_600_) was ∼0.50, and were then used for in vitro assays.

### Fluctuation Analyses

Fluctuation analyses were performed as described by Luria and Delbrück (1943). Briefly, an aliquot from the starter cultures for each strain was used to inoculate 350 ml of 7H9 ADC to have an initial bacterial density of 5,000 colony forming units (CFU) per milliliter. This was immediately divided into 33 parallel cultures, each with 10 ml of culture volume aliquoted into individual 50 ml Falcon Conical Centrifuge Tubes (Corning Inc.). The parallel cultures were incubated at 37 °C on standing racks, with resuspension by vortexing (Bio Vortex V1, Biosan) every 24 h. Cultures were grown until an OD_600_ of between 0.40 and 0.65. Once at this density, final cell counts (*N*_t_) from three randomly chosen parallel cultures were calculated by serial dilution and plating on Middlebrook 7H11 (BD), supplemented with oleic acid (AppliChem), albumin, and catalase (hereafter referred to as 7H11 OADC). To calculate the number of resistant colonies (*r*), the remaining 30 parallel cultures not used for *N*_t_ determination were pelleted at 800×g for 10 min at 4 °C using the Allegra X-15R Benchtop Centrifuge (Beckmann Coulter). The supernatants were discarded, and the bacterial pellets resuspended in 300 μl of 7H9 ADC. The resuspensions were spread on 7H11 OADC plates supplemented with the relevant drug concentration (2, 4, or 8 µg/ml of ofloxacin, or 100 µg/ml STR; Sigma). Resistant colonies were observed and enumerated after 21–35 days of incubation, depending on the *Mtb* strain. The frequency of drug-resistant mutants per culture (*r*) was enumerated, and the estimated number of drug-resistance mutations per culture (*m*) was estimated from the distribution of the *r* values (*r*_dist_) using the Ma, Sarkar, Sandri-Maximum Likelihood Estimator method (MSS-MLE) (Rosche and Foster 2000). Values of *r* that were >300 were simply given a value of 300, as this would not change the precision of the calculated *m* value using the MSS-MLE method (Rosche and Foster 2000). The MSS-MLE method is also only valid for a range of *m* values between 0.3 and 20 (Rosche and Foster 2000). The frequency of drug-resistance mutations acquired per cell (*F*) per strain was then calculated by dividing the calculated *m* values by their respective *N*_t_ values. The 95% confidence intervals for each *F* were calculated as previously described by Rosche and Foster (2000). Hypotheses testing for significant differences between the *r*_dist_ between strains for the fluctuation analyses at 4 µg/ml of OFX (fig. 1*A*) and at 100 µg/ml of STR (fig. 2) were performed using the Kruskal–Wallis test; significant differences in the *r*_dist_ between strains in the fluctuation analyses at 2 and 8 µg/ml (fig. 1*B*) were tested for using the Wilcoxon rank-sum test. Statistical analyses were performed using the R statistical software (v.3.5.1) (R Core Team 2018).

### Determining the Mutational Profile for OFX-R In Vitro

From the parallel cultures plated on 4 µg/ml of OFX (fig. 1*A*), up to 120 resistant colonies per strain (at least 1 colony per plated parallel culture if colonies were present, to a maximum of 6) were transferred into 100 μl of sterile deionized H_2_O placed in Falcon 96-well Clear Microplate (Corning Inc.). The bacterial suspensions were then heat-inactivated at 95 °C for 1 h, and used as PCR templates to amplify the QRDR in *gyrA* and *gyrB* using primers designed by Feuerriegel et al. (2009). PCR products were sent to Macrogen, Inc. or Microsynth AG for Sanger sequencing, and QRDR mutations were determined by aligning the PCR product sequences against the H37Rv reference sequence (Cole et al. 1998). Sequence alignments were performed using the Staden Package (Staden 1996), while the amino acid substitutions identification were performed using the Molecular Evolutionary Genetics Analysis Version 6.0 package (Tamura et al. 2013). Fisher’s exact test was used to test for significant differences between the strains’ mutational profiles for OFX-R. Data analyses were performed using the R statistical software (v.3.5.1) (R Core Team 2018), and figures were produced using the ggplot2 package (Wickham, 2016).

### Isolation of Spontaneous Ofloxacin-Resistant Mutants

Spontaneous OFX-resistant mutants were isolated from strains belonging one of three genetic backgrounds: N0157 (L1, Manila sublineage), N1283 (L4, Ural sublineage), and N0145 (L2, Beijing sublineage) (Borrell et al. 2019). To begin, we transferred 50 μl of starter cultures for each strain into separate culture tubes containing 10 ml of fresh 7H9 ADC. Cultures were incubated at 37 °C until OD_600_ of ∼0.80, and pelleted at 800×g for 5 min at 4 °C. The supernatant was discarded, and the pellet resuspended in 300 μl of 7H9 ADC. The resuspension was plated on 7H11 OADC (BD) supplemented with 2 μg/ml of OFX, and incubated until resistant colonies appeared (∼14–21 days). Resistant colonies were picked and resuspended in fresh 10 ml 7H9 ADC, and incubated at 37 °C. Once the culture reached early stationary phase, two aliquots were prepared. The first aliquot was heat-inactivated at 95 °C for 1 h, and the *gyrA* mutation identified by PCR and Sanger sequencing, as described in the mutational profile for OFX-R assay. If the first aliquot harbored one of four OFX-R *gyrA* mutations (GyrA^D94G^, GyrA^D94N^, GyrA^A90V^, or GyrA^G88C^), the second aliquot was stored in −80 °C for future use.

Prior to further experimentation with the spontaneously OFX-resistant mutant strains, starter cultures were prepared in the same manner as for the drug-susceptible strains.

### Drug Susceptibility Assay

We determined the OFX-susceptibility levels of our spontaneous OFX-resistant mutants and their respective drug-susceptible ancestors by performing the colorimetric, microtiter plate-based Alamar Blue assay (Franzblau et al. 1998). Briefly, we used a Falcon 96-well Clear Microplate, featuring a serial 2-fold dilution of OFX. For drug-susceptible strains, a range of OFX concentration from 15 to 0.058 μg/ml was used. Meanwhile, for OFX-resistant strains, a range of 60–0.234 μg/ml was used. Each well was inoculated with a 10 μl volume of starter culture to have a final inoculum of ∼5 × 10^6^ CFU/ml. The plates were incubated at 37 °C for 10 days. Following incubation, 10 μl of Resazurin (Sigma) were added to each well, and the plates were incubated for another 24 h at 37 °C. After this incubation period, plates were inactivated by adding 100 μl of 4% formaldehyde to every well. Measurement of fluorescence produced by viable cells was performed on SpectraMAX GeminiXPS Microplate Reader (Molecular Devices). The excitation wavelength was set at 536 nm, and the emission wavelength at 588 nm was measured. Minimum inhibitory concentration (MIC) for OFX was determined by first fitting a Hill curve to the distribution of fluorescence, and then defining the MIC as the lowest OFX concentration where the fitted Hill curve showed a ≥ 95% reduction in fluorescence. Two sets of experiments were performed for every strain, with three technical replicates per experiment. Analyses of MIC data were performed and figures created using the numpy, scipy, pandas, and matplotlib modules for the Python programming language.

### Cell Growth Assay

We set up three or four 1,000 ml roller bottles with 90 ml of 7H9 ADC and 10 ml borosilicate beads. Each bottle was inoculated with a volume of starter cultures so that the initial bacterial density was at an OD_600_ of 5 × 10^−7^. The inoculated bottles were then placed in a roller incubator set to 37 °C, and incubated for 12–18 days with continuous rolling. OD_600_ measurements were taken once or twice every 24 h. Two independent experiments in either triplicates or quadruplicates were performed per strain.

We defined the exponential phase as the bacterial growth phase where we observed a log_2_-linear relationship between OD_600_ and time; specifically, we used a Pearson’s *R*^2^ value ≥ 0.98 as the threshold. The growth rate of a particular strain was then defined as the slope of the linear regression model. The relative fitness of a given spontaneous OFX-R mutant was defined by taking the growth rate of the OFX-resistant mutant strain and dividing it by the growth rate of its respective drug-susceptible ancestor. Linear regression models for the cell growth assays data were performed using the numpy, scipy, pandas, and matplotlib modules for the Python programming language, as well as the R statistical software (v.3.5.1) and the ggplot2 package (Wickham, 2016; R Core Team 2018).

### Surveying the FQ-R Profile from Publicly Available *Mtb* Genomes

We screened public databases to download global representatives of *Mtb* genomes, as described by Menardo et al. (2018). We selected genomes that were classified as MDR-TB based on the presence of both INH-R and RIF-R mutations. This provided a data set of 3,450 genomes with confirmed MDR-TB; their accession numbers are reported in supplementary table S8, Supplementary Material online. These MDR-TB genomes were then screened for the presence of FQ-R mutations, and we identified 854 genomes that were classified as FQ-R.

The INH-R, RIF-R, and FQ-R mutations used for screening are the same mutations used by Payne et al. (2019), and are listed in supplementary table S13, Supplementary Material online. A drug-resistance mutation was defined as “fixed” in the population when it reached a frequency of ≥90%. Meanwhile, a drug-resistance mutation was considered “variable” in the population when its frequency was between 10% and 90%; thus, multiple drug-resistance mutations may be present in the genomic data from a single *Mtb* clinical isolate.

### Defining Transmission Clusters and Determining the Frequency of FQ-R *gyrA* Mutation Events

To define transmission clusters, the differences in the number of SNPs were used as a measure of genetic distance between two *Mtb* genomes. Using the haplotypes package (v.1.0) for the R statistical software (v.3.5.1) (R Core Team 2018), a genetic distance matrix was then inferred for the 3,450 MDR-TB genomes. Insertions/deletions were considered missing data. Agglomerative clustering was performed using the agnes function from the cluster package (v.2.0.6) for the R statistical software (R Core Team 2018). A conservative threshold of 12 SNPs average distance was used to define likely patient-to-patient transmission (Walker et al. 2013), and the tree was cut at a height of 12 SNPs using the hclust function. All resulting transmission clusters, with a minimum size of two clustered genomes, were used for further analysis. For every transmission cluster, each unique and fixed FQ-R *gyrA* mutation was treated as an independent mutation event. Fixed FQ-R *gyrA* mutations in nonclustered *Mtb* genomes were also treated as an independent mutation event. Figures were produced using the ggplot2 package (Wickham, 2016). 

## Supplementary Material

msz214_Supplementary_Data
