# Supplementary material for: The Genetic Background Modulates the Evolution of Fluoroquinolone-Resistance in Mycobacterium tuberculosis
Source: Mol Biol Evol. 2019 Sep 18;37(1):195–207. doi: 10.1093/molbev/msz214 (PMC6984360; doi:10.1093/molbev/msz214)
Supplement: msz214_Supplementary_Data [file msz214_supplementary_data.zip › msz214-Suppl_data/Supplementary_Data.pdf]

Supplementary Material for:

The Genetic Background modulates the Evolution of Fluoroquinolone-Resistance in *Mycobacterium tuberculosis*

Rhastin A. D. Castro[1,2], Amanda Ross[1,2], Lujeko Kamwela[1,2], Miriam Reinhard[1,2], Chloé Loiseau[1,2], Julia Feldmann[1,2], Sonia Borrell[1,2], Andrej Trauner[1,2,†], and Sebastien Gagneux[1,2,*]

[1]Swiss Tropical and Public Health Institute, Basel, Switzerland

[2]University of Basel, Basel, Switzerland

[*,†]Corresponding authors

Socinstrasse 57, 4051 Basel, Switzerland

T: +41 61 284 6983

F: +41 61 284 8101

Email: sebastien.gagneux@swisstph.ch

Email: andrej.trauner@swisstph.ch

This PDF file includes:

Supplementary Figs. S1 to S5

Supplementary Tables S1 to S13

References for Supplementary Material citations

Supplementary Figures

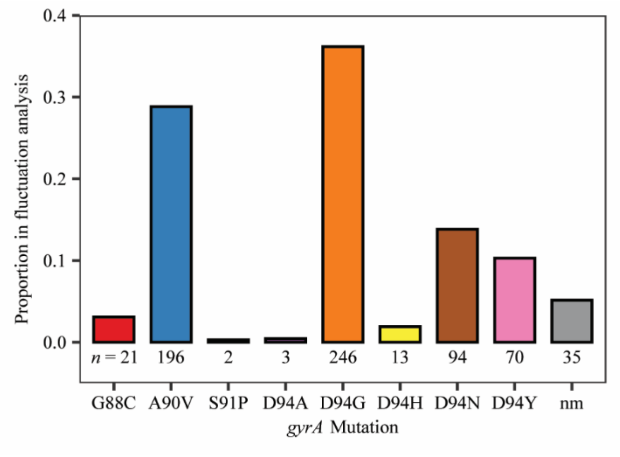

Supplementary Fig. S1

Proportion of each *gyrA* mutation after sequencing of the QRDR of *gyrA* in 680 ofloxacin-resistant colonies from the fluctuation analysis performed in Fig. 2A (nm = no identified QRDR *gyrA* mutations). The numbers of colonies with the given *gyrA* mutation are reported directly below each respective column.

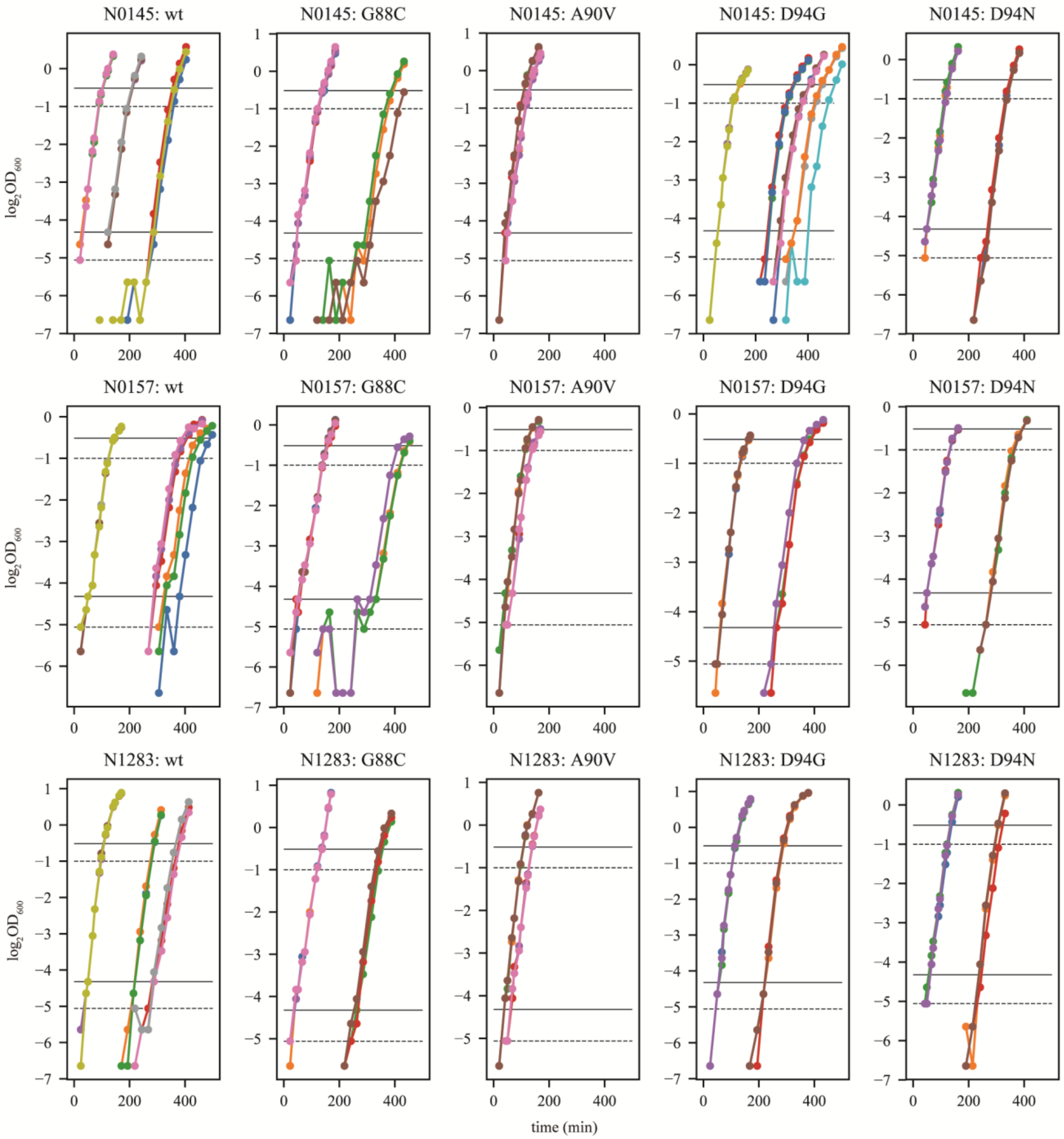

Supplementary Fig. S2

Growth profiles of *M. tuberculosis* strains in cell growth assays under antibiotic free conditions, with all OD_{600} values plotted (log_2-transformed). Genetic background of *M. tuberculosis* strain and

its corresponding *gyrA* mutation are presented above each respective plot. Coloured dots represent the measured log_2OD_{600} values at a given time (in minutes), with coloured lines connecting respective coloured dots. Different colours represent different replicates for each strain. For reference, black solid horizontal lines were plotted to denote non-transformed OD_{600} values of either 0.05 (lower line) or 0.70 (upper line), while black dashed lines denote OD_{600} values of either 0.03 (lower line) or 0.50 (upper line).

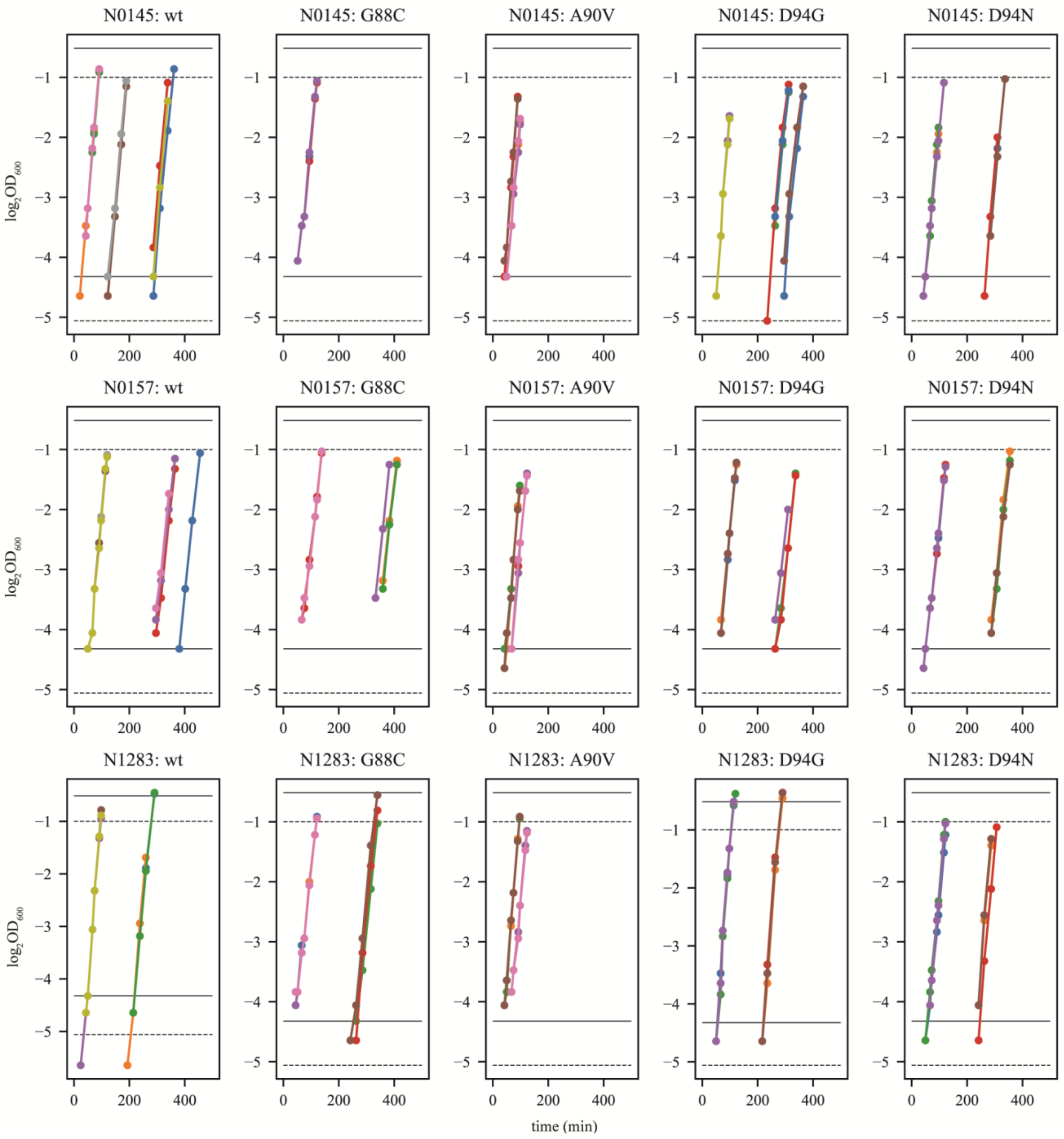

Supplementary Fig. S3

Growth profiles of *M. tuberculosis* strains in cell growth assays under antibiotic free conditions, with only measured OD_{600} values (log_2-transformed) present after filtering for exponential phase of growth. Genetic background of *M. tuberculosis* strain and its corresponding *gyrA* mutation are

presented above each respective plot. Exponential growth phase was defined as a set consecutive time-points where a linear relationship between $\log_2 OD_{600}$ (defined by a Pearson's R^2 value ≥ 0.98) and time was present. Coloured dots represent the measured $\log_2 OD_{600}$ values at a given time (in minutes), with coloured lines connecting respective coloured dots. Different colours represent different replicates for each strain. For reference, black solid horizontal lines were plotted to denote non-transformed OD_{600} values of either 0.05 (lower line) or 0.70 (upper line), while black dashed lines denote OD_{600} values of either 0.03 (lower line) or 0.50 (upper line).

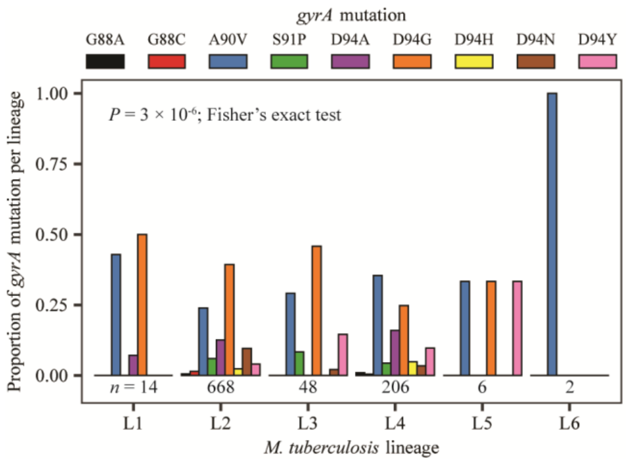

Supplementary Fig. S4

Mutational profile for all (fixed and variable) fluoroquinolone-resistance *gyrA* mutations is lineage-specific in clinical isolates of *M. tuberculosis*. An initial dataset of 3,450 genomes with confirmed MDR-TB mutations were surveyed. 854 genomes were identified as fluoroquinolone-resistant, with 848 of these genomes containing *gyrA* mutations. If genomic data from a single *M. tuberculosis* clinical isolate contained multiple fluoroquinolone-resistance *gyrA* mutation, each mutation was counted once (*n* = 944). Number of genomes analyzed per lineage is presented underneath each respective bar graph. *Mtb* lineage designations defined as in Comas et al. 2010; Gagneux 2018.

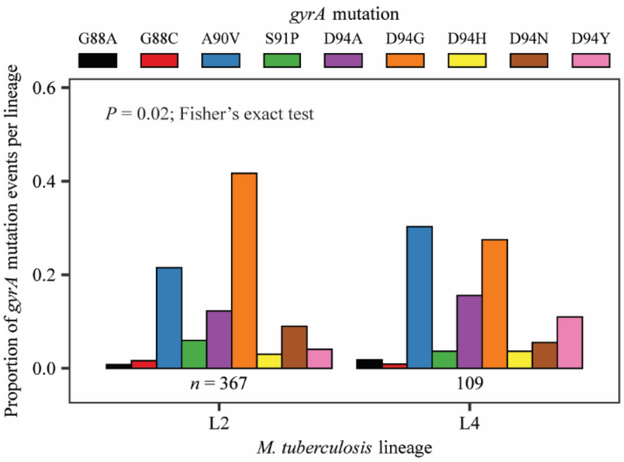

Supplementary Fig. S5

Variation in the frequency of mutation events per fluoroquinolone-resistance (FQ-R) *gyrA* mutation amongst clinical isolates of *M. tuberculosis* belonging to either L2 or L4 lineages. Mutation events per FQ-R *gyrA* mutation were enumerated from an initial dataset of 3,450 genomes with confirmed MDR-TB mutations. Genomes were defined as belonging to a given transmission cluster by using a pairwise genetic distance threshold of 12 single nucleotide polymorphisms average as a cut-off. Each unique and fixed FQ-R *gyrA* mutation present per transmission cluster, as well as each fixed FQ-R *gyrA* mutation present in non-clustered genomes, were counted as independent mutation events. The number of FQ-R *gyrA* mutation events per lineage is presented underneath each respective bar graph. *Mtb* lineage designations defined as in Comas et al. 2010; Gagneux 2018.

Supplementary Tables

Supplementary Table S1

Classification of *M. tuberculosis* strains used for *in vitro* assays

Strain	Lineage	Sub-lineage	Alternate Sub-lineage/Strain Nomenclatures
N0069	L1	L1.1.1	EAS042 (Hershberg et al. 2008)
N0072	L1	L1.1.2	EAS053 (Hershberg et al. 2008)
N0157	L1	L1.2.1	Manila; T92 (Tsolaki et al. 2004)
N0052	L2	L2.2.2	Beijing, Asia Central 1; 98_1833 (Hershberg et al. 2008; Coll et al. 2014; Shitikov et al. 2017)
N0145	L2	L2.2.1.1	Beijing, Pacific RD150; T67 (Tsolaki et al. 2004; Coll et al. 2014; Shitikov et al. 2017)
N0155	L2	L2.2.1	Beijing; T85 (Tsolaki et al. 2004; Gagneux et al. 2006)
N0136	L4	L4.3.3	Latin America-Mediterranean; T4 (Tsolaki et al. 2004; Coll et al. 2014; Stucki et al. 2016)
N1216	L4	L4.6.2.2	Cameroon (Coll et al. 2014; Stucki et al. 2016)
N1283	L4	L4.2.1	Ural (Coll et al. 2014; Stucki et al. 2016)

Lineage 1 = L1; Lineage 2 = L2; Lineage 4 = L4. *Mtb* lineage designations defined as in Comas et al. 2010; Gagneux 2018.

Supplementary Table S2

Phylogenetic single nucleotide polymorphisms leading to missense DNA gyrase or DnaE mutations that are present in the genomic data of the nine drug-susceptible *M. tuberculosis* strains outlined in Supplementary Table S1

Gene	Gene Name	Amino Acid Substitution	Strain	Lineage
Rv0005	*gyrB*	M291I	N0069	L1
Rv0005	*gyrB*	M291I	N0072	L1
Rv0005	*gyrB*	M291I	N0157	L1
Rv0006	*gyrA*	A384V	N0069	L1
Rv0006	*gyrA*	A384V	N0072	L1
Rv0006	*gyrA*	A384V	N0157	L1
Rv0006	*gyrA*	G247S	N0136	L4
Rv0006	*gyrA*	K224E	N0072	L1
Rv0006	*gyrA*	P154R	N1216	L4
Rv1547	*dnaE1*	D316N*	N0069	L1
Rv1547	*dnaE1*	S898L*	N0052	L2
Rv3370c	*dnaE2*	C313W*	N0157	L1
Rv3370c	*dnaE2*	P814S*	N0052	L2

*These mutations do not occur in the polymerase and histidinol phosphatase-domain of DNA polymerase *dnaE*, and therefore are not confirmed to confer a hypermutator phenotype in *M. tuberculosis* (Rock et al. 2015; Baños-Mateos et al. 2017)

Supplementary Table S3

Mutations present in the *rpsL* gene for 194 streptomycin-resistant colonies following fluctuation analysis on 100 µg/mL of streptomycin

Strain	K43M	K43N	K43R	K43R, K10Q	K43T	K88E	K88R	nm
N0157	0	1	5	0	9	0	0	0
N1283	4	4	72	1	13	3	5	9
N0145	0	0	46	0	13	0	0	9

nm = streptomycin-resistant colonies with no mutations in *rpsL*

Supplementary Table S4

Mutations in the QRDR of *gyrA* for 680 ofloxacin-resistant colonies following fluctuation analysis on 4 µg/mL of ofloxacin

Strain	G88C	A90V	S91P	D94A	D94G	D94H	D94N	D94Y	nm	Strain Total
N0157	1	43	0	0	9	1	7	1	2	64
N0072	2	112	0	2	37	0	1	11	7	172
N0052	13	4	0	0	39	2	18	15	21	112
N0155	2	0	0	0	51	0	14	14	3	84
N1283	1	12	1	0	36	7	14	19	0	90
N0136	0	24	1	1	31	3	13	9	0	82
N1216	1	0	0	0	27	0	16	1	0	45
N0069	1	1	0	0	13	0	4	0	2	21
N0145	0	0	0	0	3	0	7	0	0	10
**Mutation Total**	21	196	2	3	246	13	94	70	35	680

nm = ofloxacin-resistant colonies with no mutations in the QRDR region of *gyrA*. Strains are ordered top to bottom based on their frequency of OFX-resistance at 4 µg/mL OFX as shown in Figure 1A.

Supplementary Table S5

Mutations in the QRDR of *gyrB* for 590 ofloxacin-resistant colonies following fluctuation analysis on 4 μg/mL of ofloxacin

**Strain**	**E454K**	**D461H**	**nm**	**Strain Total**
N0157	0	1	41	22
N0072	0	0	155	155
N0052	1	0	101	102
N0155	0	0	66	7
N1283	0	0	69	46
N0136	0	0	81	69
N1216	0	0	46	81
N0069	0	0	22	42
N0145	0	0	7	66
**Mutation Total**	1	1	588	590

nm = ofloxacin-resistant colonies with no mutations in the QRDR region of *gyrB*. Strains are ordered top to bottom based on their frequency of OFX-resistance at 4 μg/mL OFX as shown in Figure 1A.

Supplementary Table S6

Ofloxacin MIC estimates for *gyrA* mutant strains and their respective parental strain

Strain	*gyrA* Mutation	Genetic Background (Parental Strain)	Ofloxacin MIC (µg/mL)	Normalized Ofloxacin MIC*
N0157	wt	---	2.00	1.00
N3661	G88C	*N0157*	31.60	15.80
N2034	A90V	*N0157*	10.00	5.00
N2036	D94G	*N0157*	20.00	10.00
N2035	D94N	*N0157*	20.00	10.00
N1283	wt	---	0.60	1.00
N2508	G88C	*N1283*	12.60	21.00
N2505	A90V	*N1283*	4.00	6.67
N3915	D94G	*N1283*	12.60	21.00
N2507	D94N	*N1283*	12.60	21.00
N0145	wt	---	0.50	1.00
N3659	G88C	*N0145*	39.80	79.60
N2847	A90V	*N0145*	3.20	6.40
N1893	D94G	*N0145*	10.00	20.00
N1895	D94N	*N0145*	10.00	20.00

MIC estimates for Ofloxacin based on fitting of a Hill curve to the distribution of fluorescence in an Alamar Blue assay (Franzblau et al. 1998). MIC is defined as the ofloxacin concentration where fitted Hill curve showed a ≥95% reduction in fluorescence. *Normalized Ofloxacin MIC is calculated by taking the ofloxacin MIC of a given *M. tuberculosis* strain and dividing it by the ofloxacin MIC of its respective wild-type parental strain; Normalized Ofloxacin MICs for each wild-type parental strain are therefore equal to 1.00.

Supplementary Table S7

***In vitro* fitness of *M. tuberculosis* strains based on cell growth assays in antibiotic-free conditions**

**Strain**	***gyrA* Mutation**	**Genetic Background (Parental Strain)**	**Growth Rate (GR)**	**GR: Lower 95%**	**GR: Upper 95%**	**Generation Time (in hours)**	**Relative Fitness (RF)**	**RF: Lower 95%**	**RF: Upper 95%**	***P***
N0157	wt	---	0.045	0.044	0.047	22.22				
N3661	G88C	*N0157*	0.038	0.034	0.042	26.36	0.844	0.773	0.894	**<0.001***
N2034	A90V	*N0157*	0.052	0.048	0.057	19.23	1.156	1.091	1.213	**<0.001***
N2036	D94G	*N0157*	0.044	0.04	0.048	22.73	0.978	0.909	1.021	0.354
N2035	D94N	*N0157*	0.042	0.038	0.046	23.81	0.933	0.864	0.979	**0.009***
N1283	wt	---	0.061	0.059	0.064	16.40				
N2508	G88C	*N1283*	0.042	0.037	0.047	23.81	0.689	0.627	0.734	**<0.001***
N2505	A90V	*N1283*	0.054	0.048	0.06	18.52	0.885	0.814	0.938	**<0.001***
N3915	D94G	*N1283*	0.062	0.056	0.068	16.13	1.016	0.949	1.062	0.638
N2507	D94N	*N1283*	0.052	0.046	0.058	19.23	0.852	0.780	0.906	**<0.001***
N0145	wt	---	0.053	0.05	0.055	18.87				
N3659	G88C	*N0145*	0.044	0.038	0.05	22.73	0.830	0.760	0.909	**<0.001***
N2847	A90V	*N0145*	0.058	0.051	0.063	17.24	1.094	1.020	1.145	**0.016***
N1893	D94G	*N0145*	0.05	0.044	0.056	20.00	0.943	0.880	1.018	0.107
N1895	D94N	*N0145*	0.051	0.044	0.056	19.61	0.962	0.880	1.018	0.206

The growth rate of a particular *M. tuberculosis* strain was defined as the slope during exponential phase of bacterial growth, with the exponential phase of bacterial growth defined as where a $\log_2$-linear relationship existed between OD_{600} and time using a Pearson's R^2 value ≥ 0.98 as the threshold. Generation times were calculated by taking the inverse of the calculated growth rate. The relative fitness of a given *gyrA* mutant was defined by taking its growth rate and dividing it by the growth rate of its respective wild-type ancestor.

Supplementary Table S8

Accession number of genomes used from *M. tuberculosis* clinical isolates with confirmed MDR-TB mutations

(attached as a separate TSV file due to excessive length; *n* = 3,450)

Supplementary Table S9

Number of publicly available genomes from *M. tuberculosis* clinical isolates used to survey the mutational profile for fluoroquinolone-resistance

***Mtb* lineage**	**No. of MDR-TB Genomes**	**No. of MDR-TB + FQ-R Genomes**
Lineage 1	109	13
Lineage 2	1,903	597
Lineage 3	151	44
Lineage 4	1,261	196
Lineage 5	18	2
Lineage 6	8	2
**Total**	3,450	854

Mtb = *M. tuberculosis*; MDR-TB = multidrug-resistant tuberculosis, defined as *Mtb* genomes that have both an isoniazid and a rifampicin-resistance mutation; FQ-R = fluoroquinolone-resistant, defined as *Mtb* genomes that have a fluoroquinolone-resistance mutation. Multiple drug-resistance mutations present in the genomic data from a single *Mtb* clinical isolate is possible (classified as "variable," and therefore not "fixed" for drug-resistance mutations); if a genome contained multiple drug-resistance mutations, then the genome is simply counted as MDR-TB or FQ-R once. *Mtb* lineage designations defined as in Comas et al. 2010; Gagneux 2018.

Supplementary Table S10

Frequency of all (fixed and variable) fluoroquinolone-resistance mutations from sample set of 3,450 MDR-TB genomes.

**Mutation**	**L1**	**L2**	**L3**	**L4**	**L5**	**L6**	**Mutation Total**
gyrA G88A	0	4	0	2	0	0	6
gyrA G88C	0	10	0	1	0	0	11
gyrA A90V	6	160	14	73	2	2	257
gyrA S91P	0	40	4	9	0	0	53
gyrA D94A	1	84	0	33	0	0	118
gyrA D94G	7	263	22	51	2	0	345
gyrA D94H	0	16	0	10	0	0	26
gyrA D94N	0	64	1	7	0	0	72
gyrA D94Y	0	27	7	20	2	0	56
gyrB D461N	0	3	0	2	0	0	5
gyrB N499D	0	1	0	0	0	0	1
**Lineage Total**	14	672	48	208	6	2	950

If a genome was classified as "variable" for fluoroquinolone-resistance mutations, each *gyrA* or *gyrB* mutation present was counted once. Lineage 1 = L1, Lineage 2 = L2, Lineage 3 = L3, Lineage 4 = L4, Lineage 6 = L5, Lineage 6 = L6. *Mtb* lineage designations defined as in Comas et al. 2010; Gagneux 2018.

Supplementary Table S11

Frequency of fixed fluoroquinolone-resistance mutations from sample set of 3,450 MDR-TB genomes.

**Mutation**	**L1**	**L2**	**L3**	**L4**	**L5**	**L6**	**Mutation Total**
gyrA G88A	0	3	0	2	0	0	5
gyrA G88C	0	8	0	1	0	0	9
gyrA A90V	4	114	9	61	0	2	190
gyrA S91P	0	26	4	7	0	0	37
gyrA D94A	0	70	0	26	0	0	96
gyrA D94G	6	200	16	39	0	0	261
gyrA D94H	0	12	0	4	0	0	16
gyrA D94N	0	48	1	6	0	0	55
gyrA D94Y	0	19	5	17	0	0	41
gyrB D461N	0	3	0	2	0	0	5
gyrB N499D	0	1	0	0	0	0	1
**Lineage Total**	10	504	35	165	0	2	716

Only genomes classified as "fixed" for fluoroquinolone-resistance mutations were enumerated here. Notably, no "fixed" mutations were observed in Lineage 5 (L5) strains. Lineage 1 = L1, Lineage 2 = L2, Lineage 3 = L3, Lineage 4 = L4, Lineage 6 = L6. *Mtb* lineage designations defined as in Comas et al. 2010; Gagneux 2018.

Supplementary Table S12

Frequency of mutation events per fluoroquinolone-resistance *gyrA* mutation from an initial sample set of 3,450 MDR-TB genomes.

**Mutation**	**L1**	**L2**	**L3**	**L4**	**L5**	**L6**	**Mutation Events Total**
gyrA G88A	0	3	0	2	0	0	5
gyrA G88C	0	6	0	1	0	0	7
gyrA A90V	4	79	9	33	0	2	127
gyrA S91P	0	22	3	4	0	0	29
gyrA D94A	0	45	0	17	0	0	62
gyrA D94G	3	153	15	30	0	0	201
gyrA D94H	0	11	0	4	0	0	15
gyrA D94N	0	33	1	6	0	0	40
gyrA D94Y	0	15	3	12	0	0	30
**Lineage Total**	7	367	31	109	0	2	516

Only “fixed” fluoroquinolone-resistance *gyrA* mutation events were enumerated here. Notably, no “fixed” mutations were observed in Lineage 5 (L5) strains. Lineage 1 = L1, Lineage 2 = L2, Lineage 3 = L3, Lineage 4 = L4, Lineage 6 = L6. *Mtb* lineage designations defined as in Comas et al. 2010; Gagneux 2018.

Supplementary Table S13

List of high-confidence drug-resistance mutations used to determine drug-resistance mutational profiles of genomes from clinical isolates of *M. tuberculosis*

(attached as a CSV file due to length; n = 196)

References

Baños-Mateos S, Roon A-MM van, Lang UF, Maslen SL, Skehel JM, Lamers MH. 2017. High-fidelity DNA replication in *Mycobacterium tuberculosis* relies on a trinuclear zinc center. Nat. Commun. 8:1–10.

Coll F, McNerney R, Guerra-Assunção JA, Glynn JR, Perdigão J, Viveiros M, Portugal I, Pain A, Martin N, Clark TG. 2014. A robust SNP barcode for typing *Mycobacterium tuberculosis* complex strains. Nat. Commun. 5:4812.

Comas I, Chakravartti J, Small PM, Galagan J, Niemann S, Kremer K, Ernst JD, Gagneux S. 2010. Human T cell epitopes of *Mycobacterium tuberculosis* are evolutionarily hyperconserved. Nat. Genet. 42:498–503.

Franzblau SG, Witzig RS, McLaughlin JC, Torres P, Madico G, Hernandez A, Degnan MT, Cook MB, Quenzer VK, Ferguson RM, et al. 1998. Rapid, Low-Technology MIC Determination with Clinical *Mycobacterium tuberculosis* Isolates by Using the Microplate Alamar Blue Assay. J. Clin. Microbiol. 36:362–366.

Gagneux S. 2018. Ecology and evolution of *Mycobacterium tuberculosis*. Nat. Rev. Microbiol. 16:202–213.

Gagneux S, Long CD, Small PM, Van T, Schoolnik GK, Bohannan BJM. 2006. The Competitive Cost of Antibiotic Resistance in *Mycobacterium tuberculosis*. Science 312:1944–1946.

Hershberg R, Lipatov M, Small PM, Sheffer H, Niemann S, Homolka S, Roach JC, Kremer K, Petrov DA, Feldman MW, et al. 2008. High Functional Diversity in *Mycobacterium tuberculosis* Driven by Genetic Drift and Human Demography. PLOS Biol. 6:e311.

Rock JM, Lang UF, Chase MR, Ford CB, Gerrick ER, Gawande R, Coscolla M, Gagneux S, Fortune SM, Lamers MH. 2015. DNA replication fidelity in *Mycobacterium tuberculosis* is mediated by an ancestral prokaryotic proofreader. Nat. Genet. 47:677–681.

Shitikov E, Kolchenko S, Mokrousov I, Bespyatykh J, Ischenko D, Ilina E, Govorun V. 2017. Evolutionary pathway analysis and unified classification of East Asian lineage of *Mycobacterium tuberculosis*. Sci. Rep. 7:1–10.

Stucki D, Brites D, Jeljeli L, Coscolla M, Liu Q, Trauner A, Fenner L, Rutaihwa L, Borrell S, Luo T, et al. 2016. *Mycobacterium tuberculosis* lineage 4 comprises globally distributed and geographically restricted sublineages. Nat. Genet. 48:1535–1543.

Tsolaki AG, Hirsh AE, DeRiemer K, Enciso JA, Wong MZ, Hannan M, Salmoniere Y-OLG de la, Aman K, Kato-Maeda M, Small PM. 2004. Functional and evolutionary genomics of *Mycobacterium tuberculosis*: Insights from genomic deletions in 100 strains. Proc. Natl. Acad. Sci. 101:4865–4870.
